# Hypoxic Preconditioned Neural Stem Cell-Derived Extracellular Vesicles Contain Distinct Protein Cargo from Their Normal Counterparts

**DOI:** 10.3390/cimb45030127

**Published:** 2023-03-01

**Authors:** Tahereh Gharbi, Chang Liu, Haroon Khan, Zhijun Zhang, Guo-Yuan Yang, Yaohui Tang

**Affiliations:** Department of Biomedical Engineering, Shanghai Jiao Tong University, Shanghai 200025, China

**Keywords:** extracellular vesicles, hypoxia, neural stem cell, preconditioning, differentially expressed proteins

## Abstract

Hypoxic preconditioning has been demonstrated to increase the resistance of neural stem cells (NSCs) to hypoxic conditions, as well as to improve their capacity for differentiation and neurogenesis. Extracellular vesicles (EVs) have recently emerged as critical mediators of cell–cell communication, but their role in this hypoxic conditioning is presently unknown. Here, we demonstrated that three hours of hypoxic preconditioning triggers significant neural stem cell EV release. Proteomic profiling of EVs from normal and hypoxic preconditioned neural stem cells identified 20 proteins that were upregulated and 22 proteins that were downregulated after hypoxic preconditioning. We also found an upregulation of some of these proteins by qPCR, thus indicating differences also at the transcript level within the EVs. Among the upregulated proteins are CNP, Cyfip1, CASK, and TUBB5, which are well known to exhibit significant beneficial effects on neural stem cells. Thus, our results not only show a significant difference of protein cargo in EVs consequent to hypoxic exposure, but identify several candidate proteins that might play a pivotal role in the cell-to-cell mediated communication underlying neuronal differentiation, protection, maturation, and survival following exposure to hypoxic conditions.

## 1. Introduction

Neural stem cells (NSCs) are multipotent precursor cells that exist in special regions of the fetal and adult central nervous system (CNS). They have self-renewal ability and can generate neurons, astrocytes, and oligodendrocytes when needed [1]. Transplantation of neural stem cells has shown great potential in regenerative medicine. However, the therapeutic efficacy is often limited due to high cell death, low differentiation, and reduced function after cell transplantation. Various methods have been used to modify NSCs in order to obtain the most stable and functional NSCs after transplantation [2]. For example, preconditioning using various conditions, encapsulation, and genetic modulation of cells prior to transplantation have been tested. Recently, different preconditioning protocols using cytokines and hypoxic preconditioning (HP) have been examined [3,4]. In particular, decreased oxygen pre-treatments have been demonstrated to increase the resistance of NSCs in hypoxic and ischemic conditions [5]. Previously, it was shown that EVs are involved in preconditioning [6]. HP of NSCs has also been reported to benefit NSCs and promote neuronal cell survival, neural differentiation, and neurogenesis [5,7,8,9,10,11,12,13,14,15]. However, the mechanism by which HP exerts these benefits is still unclear.

Previous evidence has shown that almost all living cells release extracellular vesicles (EVs), including microvesicles, apoptotic bodies, and exosomes. EVs exist in a variety of sizes, contents, and origins [16]. Communication between cells via EVs is more complex than through secretion of chemokines and cytokines because of the EV packaging of the biomaterials [17,18]. Based on the genetic materials and proteins that the EVs carry, they can develop a certain capability to interact with different cells or initiate different chemical cascades within the recipient cells [19]. Indeed, it has been now shown that EVs play an important role in many biological processes in both healthy and abnormal cells [16]. Hence, we hypothesized that EVs might be involved in the HP effect on NSCs, and thus a detailed examination of their protein cargo may be useful in identifying crucial proteins that may restore the function of other brain cells or aid in their rescue after hypoxia.

## 2. Results

### 2.1. NSCs Characterization, Identification, and Differentiation

**Figure 1:**
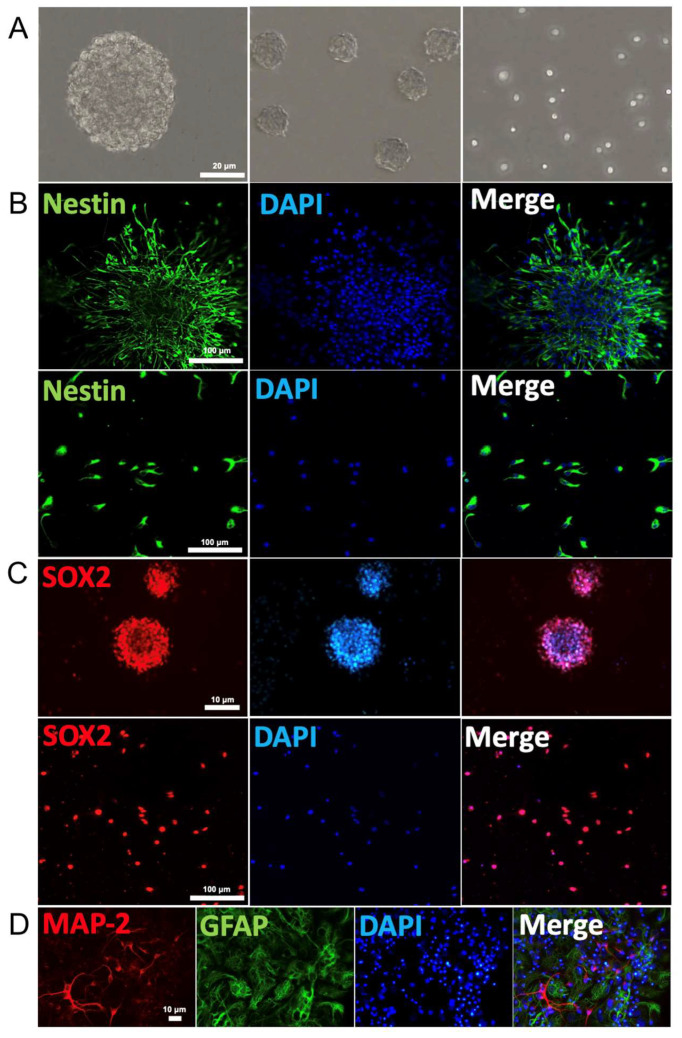
Neural stem cell identification: (**A**) Neurospheres and free single cells representing typical morphological feature of NSCs. (**B**) Immunofluorescence of neurospheres (top row) and single cells (bottom row) show expression of the NSC markers nestin (green). Cell nuclei were counterstained with DAPI (blue). (**C**) Immunofluorescence of neurospheres (top row) and single cells (bottom row) for SOX2 (red) expression and nuclei counterstained with DAPI (blue). (**D**) Immunostaining identification of the primary mouse cortex NSCs. Neural dendrites and axons were identified by anti-MAP-2 (red) and astrocytes by GFAP (green). The nuclei of all cells were identified by DAPI (blue).

We first demonstrated that NSCs cultured in growth medium proliferated into approximately uniformly sized neurospheres with typical shape as well as single cells (Figure 1A). Both neurospheres and free single cells immuno-stained positively for the stem cells markers nestin (green fluorescence) and Sox2 (red fluorescence, Figure 1B,C). These NSCs were able to differentiate into neurons and glial cells after removal of growth factor from growth cell medium over six days, as judged by the immuno-labelling of MAP-2 (red fluorescence) and GFAP (green fluorescence), respectively (Figure 1D). Thus, these results confirmed that these NSCs exhibit the typically observed biomarkers for the characterization of NSCs. Negative control was not incubated with primary antibody (Supplementary Figure S1).

### 2.2. Hypoxic Preconditioning Affects NSC Viability

**Figure 2:**
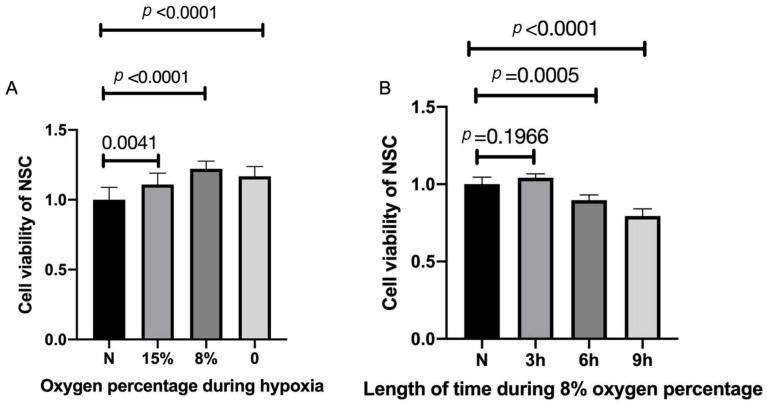
Increased cell viability after hypoxic preconditioning. (**A**) NSCs were exposed to 0%, 8%, 15%, and normal percentage of oxygen for three hours. n = 10, *p* < 0.05, each oxygen percentage group vs. normal group. (**B**) Three hours of hypoxic preconditioning of 15% and 0% oxygen exposure made no difference in cell viability, but the viability significantly increased after three hours of hypoxic preconditioning with the exposure of 8% oxygen. n = 10 cell culture wells per group, *p* < 0.05 each HP period of time group vs. normal group. Cell viabilities are all expressed as means  ±  SD.

To examine for optimal low-oxygen growth conditions, NSCs were exposed to 0%, 8%, 15%, and normal levels (20%) of oxygen for three hours. Three hours of HP of 15% oxygen exposure made a significant difference in cell viability compared with normal levels and the viability increased significantly after three hours of HP with the exposure of 8% oxygen (Figure 2A). Treatment with 0% resulted in slightly less viability than at 15%. Thus, we examined the 8% levels on cell viability for various periods of time (Figure 2B). Three hours of HP with 8% oxygen improved cell viability compared with the control group, with longer incubation periods resulting in lower viability (Figure 2B). Thus, these results suggest that 8% oxygen for three hours is optimal under our conditions to study the triggering of NSCs via HP (Figure 2B)

### 2.3. Characterization of Normal vs. Hypoxic Preconditioned NSC-Derived EVs

**Figure 3:**
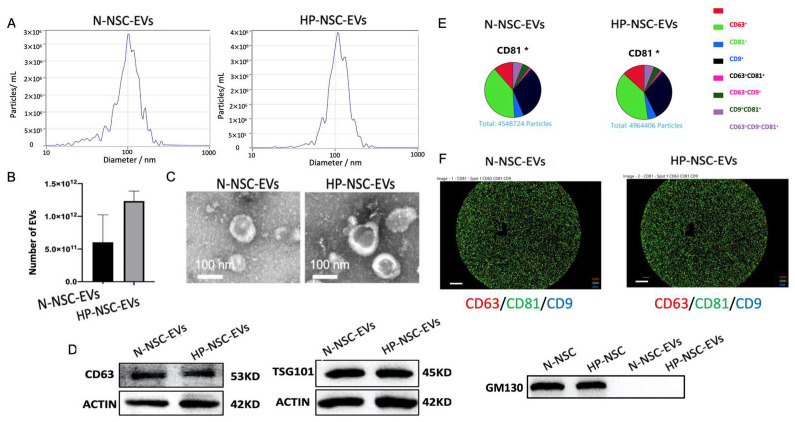
Characterization of normal vs. hypoxic preconditioned NSC-derived extracellular vesicles. (**A**). Particle size distribution measured by nanoparticle tracking analysis (NTA). (**B**) Concentration (particles/mL) of EVs measured by NTA. n = 4 NSCs isolated from mice per group, *p* = 0.0712 HP-NSC-EVs vs. N-NSC-EVs and number of EVs are expressed as means ± SD. (**C**) Normal vs. hypoxic preconditioned NSC EVs morphology identified by transmission electron microscopy (TEM). (**D**) Western blot analysis of EV surface markers, CD63, TSG101, and non-EV marker, GM130 (N = 4). (**E**) Both normal and HP-NSC-EVs were all positive for the three EV markers, CD63, CD9, and CD81. Co-expressions of EV markers were measured by probing captured EVs with the indicated secondary fluorescence-labelled antibody. (**F**) The specific EV markers from both normal and HP NSCs were analyzed by NanoSight NS300 and ExoView^®^. Scale bar = 20 μm.

NSCs were split into two groups: a group of HP incubated with 8% oxygen for 3 h and a control group kept under normal oxygen conditions. EVs were isolated from the culture medium supernatant of both groups after second passaging using ultracentrifugation. Purified NSC EVs were then characterized by nanoparticle tracking analysis (NTA), transmission electron microscopy (TEM), Western blotting, and ExoView (Figure 3A–F). NTA results demonstrated that the number of EVs was significantly greater in the HP-NSC-EVs group (Figure 3B). Co-expressions of EV markers were measured by probing captured EVs with the indicated secondary fluorescence-labelled antibody. We found that both normal and HP-NSC-EVs were all positive for the three EV markers, CD63, CD9, and CD81 (Figure 3D–F). Overall, we found that the size, morphology, and presence of surface markers were fairly similar in both groups.

### 2.4. Proteomic Profiling of Normal vs. Hypoxic Preconditioned NSC-Derived EVs

**Figure 4:**
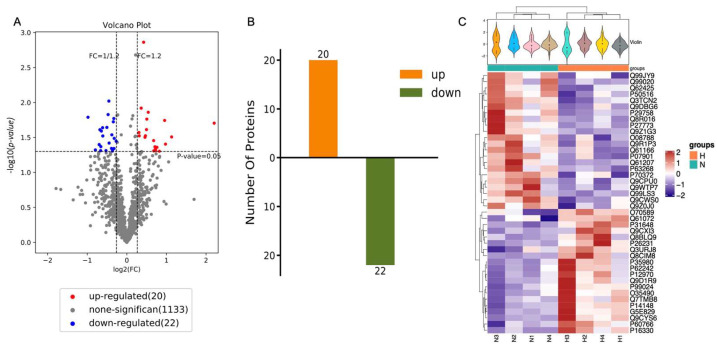
Identification of differentially expressed proteins. (**A**) Volcano differentially expressed proteins of NSC-EVs under normal condition comparing to hypoxic condition. Volcano plot indicates the differentially expressed proteins. Fold change > 1.2 or < 1/1.2 and *p* < 0.05 is considered to be a significant differentially expressed protein. Red for upregulated proteins, blue for downregulated proteins, and grey for no differentially expressed proteins. (**B**) The number of differentially expressed proteins (N-NSC-EVs vs. HP-NSC-EVs). n = 6. (**C**) Heat maps of identified proteins in N-NSC-EVs and HP-NSC-EVs.

The purified EVs from both the HP and normal group were next sequenced for their protein composition (n = 6 NSC isolated from mice per group) using the iTRAQ method. Overall, out of 2018 proteins, 42 vesicular proteins were identified to be differentially expressed: 20 proteins were identified to be overexpressed and 22 proteins were downregulated after HP (Figure 4A,B). These differences were evident by examination of each individual sample as well (Figure 4C, Table 1).

### 2.5. Differentially Expressed Proteins Enriched in Specific Biological Pathways

**Figure 5:**
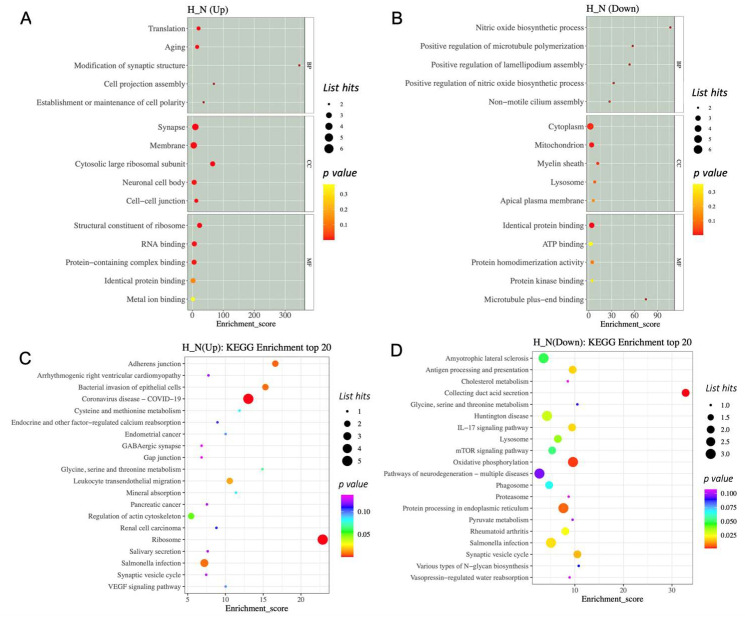
The GO and KEGG enrichment analysis of differentially expressed proteins. (**A**,**B**) GO enrichment analysis top 15 (screening the GO entries that correspond to the number of differential proteins greater than 1 in the three categories). (**C**,**D**) The KEGG enrichment analysis of differentially expressed proteins.

To get a better understanding of the protein functions and signalling pathways of hypoxia-related differentially expressed proteins (DEPs), we performed GO and KEGG pathway enrichment analysis. The top 15 enriched terms are shown in Figure 5. For the hypoxia-related upregulated DEP, the GO analysis revealed that the HP-EVs were highly enriched in translation and aging processes (Figure 5A). Further, in terms of cellular components, there was a significant enrichment of synapse, membrane, cytosolic large ribosomal subunits, neuronal cell body, and cell–cell junction complexes, while in terms of molecular functions, there was enrichment of ribosome components, as well as RNA binding and protein-containing complexes (Figure 5A). As for the hypoxia-related downregulated DEP, there was an enrichment of proteins involved in cytoplasm and mitochondrion (Figure 5A,B). In particular, for the hypoxia-related upregulated DEPs, there was an enrichment of ribosome processes as well as components involved in synaptic vesicles and cell–cell adherens and gap junctions (Figure 5D), while proteins involved in processing within the endoplasmic reticulum were enriched in the hypoxia-related downregulated DEP (Figure 5C). Interestingly, this analysis also identified proteins involved in Huntington disease, amyotrophic lateral sclerosis, and pathways of neurodegeneration among the hypoxia-related downregulated DEP. Thus, this analysis revealed a significant enrichment of specific proteins within HP-EVs that play a direct role in normal neuronal cellular functions, as well as a decrease in components known to be important in neuronal diseases.

### 2.6. Detection of Elevated mRNA Transcripts of Upregulated Proteins within HP-EVs

**Figure 6:**
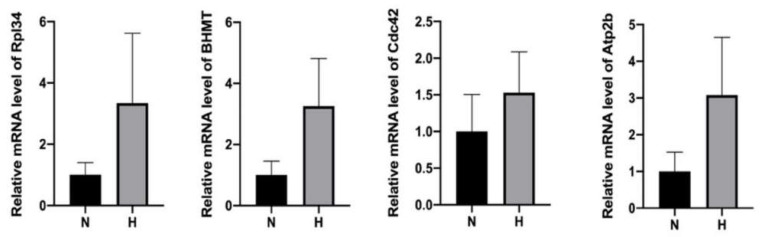
qPCR analysis revealed an enhancement of mRNA in HP-EVs of select upregulated proteins within HP-EVs. n = 4, *p* = 0.1143 HP group vs. normal group for Rpl34 mRNAs, *p* = 0.2363 HP group vs. normal group for BHMT, *p* = 0.3322 HP group vs. normal group for Cdc42, *p* = 0.1560 HP group vs. normal group for Atp2b. MRNA levels are all expressed as means ± SD.

We speculated that the presence of the upregulated proteins in the HP-EVs may be a consequence of enhanced expression in the HP-treated cells, and thus suspected that the HP-EVs might also contain an enrichment of the transcripts of these upregulated proteins as well. To test this, we determined the extent of the transcripts of select upregulated proteins by qRT-PCR in both normal and HP EVs (primer sequences are in Supplementary Table S1). Indeed, we found that the levels of mRNA of Rpl34, BHMT, Cdc42, and Atp2b were all higher in the HP-EVs exosomes than those in N-EVs (Figure 6). Thus, after the entrance of HP-EVs into recipient cells, it would result in the transfer of EV-specific proteins to recipient cells. In addition, based on our results, since the levels of some of those corresponding mRNAs also increased, the delivery of those mRNAs may also impact the protein synthesis in the recipient cells.

## 3. Discussion

EVs are well known to play important roles in both disease progression and prevention. Although some discoveries have been made, many unknown changes of the EVs under various conditions are yet poorly characterized. Based on previous studies, cells load various cargos in their EVs and release them to communicate to other cells in both balanced and imbalanced conditions. Hence, the exploration of EV cargos can help us to understand the role of EVs, their cargos, and their potential mechanisms of intercellular communication. In fact, it is highly demanding to map and compare the composition of EVs from different cells at different conditions to obtain a clear idea of the role of EVs in initiating, regulating, and suppressing diseases.

Numerous studies have been conducted regarding the RNA content of EVs. However, proteins are cardinal for final biological function; therefore, we decided to map and compare the protein cargo of NSC-EVs after HP. Despite recent efforts in understanding the role of extracellular vesicles in NSCs after hypoxia, additional studies are required to focus on the effect of hypoxia on NSC-EV secretion, production, and various signalling and biological molecules, and to distinguish the rescuing versus damaging EV cargos after hypoxia in the brain microenvironment. We successfully developed suitable conditions to induce hypoxia as a trigger for preconditioning NSCs. Previous studies demonstrated that HP could be beneficial for NSC survival, cell viability, and neurogenesis [11,20]. As previously reported, hypoxia can help maintain the proliferation and self-renewal of NSCs that could be related to an increase in HIF-1α expression [2,21,22,23]. In addition, under hypoxia, the high levels of HIF-1α elevate the level of angiogenesis-related factors such as VEGF, EPO, and GLUT which are related to neurogenesis [11].

We identified optimal conditions of 8% oxygen for 3 h to study the effects of low-oxygen exposure on NSCs (Figure 1). Previously, other studies have performed HP on NSCs with various oxygen levels and periods of time. A study found that 1% oxygen for 3 h was beneficial to NSC [12]. Similar to another study that examined preconditioning of NSCs with oxygen–glucose deprivation, 0% oxygen was found to be beneficial to NSC for 2 h and by increasing the length of time, the cell viability was lowered and cells started to be injured [20]. By contrast, in our study, our aim was to slightly trigger NSC with a lower amount of oxygen. Our result identified 8% oxygen to be the most beneficial and least devastating to NSCs. We also found that after HP, NSC increased the quantity of EV secretion, similar to many previous reports regarding various kind of cells [24].

Our work clearly showed that hypoxia influenced the protein composition within NSC-EVs. As previously reported, hypoxia influenced the efficiency of NSC treatment in various CNS diseases. We found that 42 proteins were differentially expressed after HP in NSC-EVs (Table 1). Among the 42 proteins, 20 proteins were upregulated. We also found that the transcripts of select upregulated proteins (Rpl34, Bhmt, Cdc42, and Atp2b) were also enhanced within the NSC-EVs. Interestingly, it was previously reported that BHMT could affect the nucleus to repair epigenetic control and accelerate neuroprotection [25]. Another study found that functional recovery was improved with ascorbic acid and sodium–vitamin C cotransporter 2 promoted NSCs’ migration through Cdc42 activation to facilitate F-actin assembly, which increased the therapeutic effect of ascorbic acid and NSC migration after brain injury [26]. Further, axon repair and nerve regeneration were promoted after cerebral ischemia through Netrin-1/Rac1/Cdc42 signalling pathways [25]. Acyl ghrelin improved neurite growth after oxygen glucose deficiency injury via Cdc42 [27]. We also noted that knockdown of TDP-43 in Neuro-2a cells inhibited neurite outgrowth and induced cell death. In the knockdown cells, the Rho family members RhoA, Rac1, and Cdc42 GTPases were inactivated; thus, TDP-43 may play a role in neuronal survival through protein regulation of those proteins [28]. By causing Cdc42 succinylation, ischemic buildup of succinate lowers the activity of Cdc42 GTPase, which reduces the proliferation of neural stem cells and exacerbates cerebral ischemia/reperfusion injury [29]. EGFR degradation and autophagy interact functionally during embryonic neurogenesis, and Ccd42b and ACK govern neuronal differentiation and can offer fresh insights into this interaction [30]. Wnt5a’s changed activation of Cdc42 on FIBER provides proof that the scaffold topography can affect how differently cells respond to their microenvironments [31]. N-WASP-Arp2/3 signalling-mediated development of Purkinje cell dendrites requires the upstream activator of N-WASP, Cdc42 [32]. Rat primary hippocampal neurons undergo axonogenesis in response to cdc42’s inhibition of GSK-3 activity [33]. Axon development and exocytosis are regulated by Cdc42b downstream of its activator Arhgef7 [34]. Patients with acute ischemic stroke are regularly monitored for disease progression and recurrence risk using the biomarker Cdc42 [35]. In addition to Cdc42, de novo mutations in Atp2b1 are thought to cause a monogenic form of neurodevelopmental disability, according to genetic discoveries, the probands’ overlapping phenotypes, and functional investigations [36]. Generally, Cdc42 and Atp2b may be beneficial for neuronal survival, homeostasis, and regulation [37,38,39,40,41,42,43]. Cadm2 protein, which was also one of the upregulated proteins in NSC-EVs after HP, was also reported to be involved in neuron cell–cell adhesion, molecular signalling in neurons, axon–axon interactions, and other complex sets of interactions in the nervous system [44,45,46,47]. The possibility of Rpl34 as a novel prognostic biomarker and therapeutic target for ischemic stroke was also reported previously [48,49]. Noteworthily, other overexpressed proteins such as Cnp, Cyfip1, Cask, and Tubb5 that were also shown to be overexpressed in our proteomics result are found to be involved in neuronal regulation, protection, maturation, and development [50,51,52,53,54,55,56,57,58,59,60,61,62].

Several proteins among the 22 downregulated proteins out of 42 total changes in proteins were previously reported to be involved in neurodegenerative diseases and have various functions in neurons in both healthy and disease situations.

Some of these downregulated proteins were previously tested and found to be involved in neurodegeneration and neurodegenerative diseases. Deficiency of the Npc2 protein and downregulation of ACTG2 were reported to be involved in the pathogenesis of Niemann–Pick Type C (NPC) disease [63,64]. Blmh protein is expressed in the brain and may be involved in Huntington disease [65].

Regarding Alzheimer’s disease (AD), Blmh plays role in the metabolism of homocysteine and has a connection to AD [66]. A study reported that a unique strategy to slow AD progression may be to boost DDAH1 activity in neural cells [67]. In addition, experimental autoimmune encephalomyelitis showed that increasing Ddah1 expression improved the ability to remyelinate. By modifying Ddah1 activity, these findings offer a unique therapeutic strategy for demyelinating disorders [68]. The expression of the heat shock proteins Hsp90 was significantly increased in the cells of Alzheimer’s disease patients, according to Western blot analysis [69]. Ndufa4 was also shown to be linked to mitochondrial dysfunction in the etiology of AD [70]. Alterations in Pdia3 levels also appeared to be age- and/or pathology-dependent, corroborating the ER chaperone’s involvement in AD pathology, and supporting the Pdia3 protein as a potential novel therapeutic target for the treatment of AD [71]. P-Tau protein accumulation may be influenced by decreasing Psap levels and their interactions in neurons [72]. Other studies have suggested that Psap may play a role in Alzheimer’s disease [73,74,75]. Psap has also been linked to neurotrophic and cell-damaging effects [76,77,78].

In Parkinson’s disease (PD) brain tissue samples, numerous dysregulated microRNAs have been found by recent human and animal investigations; Psmb2 is one of the downstream target proteins impacted by those dysregulated microRNAs [79]. Genetic evidence from previous studies also shows the involvement of the Psap saposin D domain in PD [80]. Dctn1 mutations may play a role in a variety of neurodegenerative illnesses, such as familial motor neuron disease, parkinsonism, and frontotemporal atrophy [81].

Various dysregulated proteins from our study were reported to be involved in amyotrophic lateral sclerosis (ALS). REEP1 cooperates with Ndufa4 in a mechanism that protects the integrity of mitochondrial complex IV, which is linked to ALS [82,83,84,85]. Bunina bodies are found in ALS, showing that Psap may also be a part of Bunina bodies. The interaction of Psap with other proteins may change how they both operate, resulting in motor neuron degeneration in ALS [86]. Several studies have linked the Dctn1 protein to ALS and Perry syndrome [87,88,89,90,91].

Atp6v1a, dctn1, Psap, and Ddah1 have also been mentioned as being involved in neurodegeneration and other neurodegenerative diseases [92,93,94,95,96].

Atp6v1a, Ndufa4, Dctn1, and Hsp90aa1 were also linked with abnormal CNS developments such as neural tube deficiency and Dandy-Walker malformation diseases [97,98,99,100].

To conclude, our work supports the idea that HP-NSC-EVs contain important proteins that contribute to the increased survival of NSCs following HP. Many of the proteins that we found were either downregulated or overexpressed in HP-EVs. Previous research showed that some of the overexpressed proteins in our study are well known to play many roles in neuronal cell function and thus may indeed be involved in neuronal survival, regulation, protection, maturation, and differentiation. Interestingly, many of the downregulated proteins in our study were previously tested to be involved in CNS degenerative diseases. Our work further underscores the potential critical role that EVs can play in changing cellular behavior as well as their usefulness as a potential target in the diagnosis of CNS diseases.

## 4. Methods and Materials

### 4.1. Primary NSC Isolation, Culture, and Passaging

Neural stem cells were obtained from the embryonic ICR mouse cortex under approval from the Institutional Animal Care and Use Committee (IACUC) of Shanghai Jiao Tong University, Shanghai, China. Primary NSCs were isolated from embryonic day 14.5 ICR mice and cultured in DMEM/F12 medium (Gibco, Carlsbad, CA, USA) containing 2% B27 (Gibco, Carlsbad, CA, USA), 20 ng/mL fibroblast growth factor (bFGF, PeproTech, Rockhill, NJ, USA), 20 ng/mL epidermal growth factor (EGF, PeproTech), 2 nM L-glutamine (Gibco), and 1% penicillin–streptomycin (Gibco) as neurospheres in incubator at 37 °C in humidified condition containing 5% CO_2_. The cultured medium was changed every 3 days and floating neurospheres were passaged every 5–7 days using acutase (Gibco). After passaging, floating or seeded single cells with the density of 1 × 10^5^ cells/mL were used for further experiments.

### 4.2. Immunohistochemistry

PFA-fixed NSC cells were washed three times with PBS for 5 min. Then, they were incubated with 0.3% Triton X-100 for 10 min and blocked with 5% bovine serum albumin for 1 h at room temperature. Afterwards, cells were incubated with primary antibodies at 4 °C overnight. The primary antibodies were used as follows: anti-GFAP antibody 1:100 (AB5804; Millipore Corporation, Danvers, MA, USA), anti-nestin (1:100, ab81462, Abcam, Cambridge, MA, USA), anti-MAP2 (1:200, MAB3418), and anti-SOX-2 (1:200 sc-365823, Santa Cruz Biotechnology, Santa Cruz, CA, USA). After rinsing the samples with PBS, the samples were then incubated with corresponding secondary antibodies for 1 h at room temperature. Nuclei were stained using 4,6-diamidino-2-phenylindole (DAPI, 1:1000; Life Technologies, Mulgrave, VIC, Australia).

### 4.3. Hypoxic Preconditioning

Cultured NSCs were exposed to various levels of oxygen concentration in a hermetically sealed chamber at 37 °C for various periods of time. After that, the cells were returned back to normal condition. NSCs cultured in normal situation of oxygen concentration level were regarded as normal control.

### 4.4. NSC Proliferation and Cell Viability Assay

NSCs were seeded in PDL-coated 96-well plates at a density of 20 × 10^4^ cell/mL, then placed in a normal condition as a control group or treated with different levels of oxygen at different periods of time for HP. The NSC cell viability was tested by the cell counting kit-8 (CCK-8, Dojindo, Kumamoto, Japan) 24 h after treatment according to the kit instructions.

### 4.5. Extracellular Vesicle Isolation

EVs were isolated from both control and hypoxia-preconditioned NSC culture supernatant using serial, sequential centrifugation, and ultracentrifugation [101].

EV protein concentration was tested by BCA protein assay (Thermo Scientific, Waltham, MA, USA).

### 4.6. Characterization of EVs

EVs were stained with 1% uranyl acetate and detected by transmission electron microscope (TEM, Thermo Scientific) for morphology characterization. EV particle number and size distribution were evaluated by nanoparticle tracking analysis (NTA, Brookhaven, NY, USA) according to the NTA device instruction.

### 4.7. Western Blotting

EV markers of CD63 and tumor of susceptibility gene 101 (TSG 101) were detected by Western blot. Western blot analysis of EV proteins of total 20 µg per sample was conducted as previously described [101]. The primary antibodies used were:

Anti-TSG-101 (1:1000, ab125011, Abcam), anti-β-actin (1:5000, 60008, Proteintech, Chicago, IL, USA), and anti-CD63 (1:1000, sc-5275, Santa Cruz Biotechnology).

### 4.8. EV Analysis with ExoView

The ExoView Tetraspanin chip (ExoView, Boston, MA, USA) was used to identify EVs. It was arrayed with antibodies against the proteins CD81, CD63, and CD9. As a negative control, mouse IgG1 was utilized. The chip surface received 35 µL of sample, which was then incubated for a whole night. After being washed, the chips were exposed to ExoView Tetraspanin Labelling ABs (EV-TC-AB-01), which included CD9/ALEXA 488, CD81/ALEXA 555, and CD63/ALEXA 647, for co-localization tests to identify EVs. The ExoView R100 reader (ExoView) was then used to image the chips utilizing the single particle interferometric reflectance imaging sensor (SP-IRIS) technology and ExoScan v0.998 (ExoView) acquisition software. With sizing thresholds set between 50 and 200 nm in diameter, the data were examined using ExoViewer v0.998.

### 4.9. Extracellular Vesicle Proteome Profiling

Both groups of normal and HP-NSC-EVs were sampled for protein extraction and sequencing. The samples underwent tandem MS and liquid chromatography analysis after being labelled with iTRAQ labelling reagents (ABSCIEX, 4,381,663). Relative iTRAQ quantification and protein analysis were carried out by Shanghai OE Biotechnology Limited Company. The basic process of bioinformatics analysis for proteomics is to search the database for qualitative and quantitative data and then, after quality assessment and preprocessing, perform expression level analysis and functional analysis, respectively. Functional annotator analysis of identified proteins was performed using multiple common databases. GO and KEGG analyses were performed on the screened differential proteins. At the same time, correlation analysis, expression pattern clustering heat map, venn analysis, etc., were performed on the data of the differential comparison group. In addition, according to the data situation, the relevant or interesting parts were explored, such as selecting the key proteins and their functions or pathways [102].

### 4.10. EV RNA Isolation

Total RNA was extracted from both control and HP-purified NSC EVs using miRNeasy Mini kit (Qiagen, Hilden, Germany), Kit. Extracted EVs were washed and centrifuged by buffer XBP and XWP, and mixed with suitable volume of QIAzol lysate stored at room temperature for 5 min. Chloroform was added according to the protocol to separate lysate, then the upper aqueous layer was transferred to a new collection tube. After addition of proper volume of 100% ethanol, the mixture was transferred into RNAeasy MinElute spin column in a collection tube. After centrifugation, RNA in the membrane was washed with buffer RWT and PRE. DNAase/RNAse-free water was added and centrifuged at 12,000× *g* for 5 min to dilute the RNA in the collection tube. The concentration and purity of EV RNAs were checked by Thermo micro spectrophotometer NanoDrop2000.

### 4.11. RT-qPCR

EV RNA was transcribed into cDNA-containing mixtures (10 μL) by miRCURY SYBER green PCR Kit (Qiagen, GER) according to the kit instructions. Then, SYBR Green Master Mix (11203ES08, Yeason, Shanghai, China) was used to perform real-time PCR. A two-stage amplification reaction was carried out under 95 °C for 5 min, followed by 40 cycles at 95 °C for 10 s, and at 60 °C for 30 s.

### 4.12. Statistical Analysis

All information was reported as mean and standard deviation (SD). The unpaired Student’s *t* test was used to compare means between two groups, and one-way ANOVA followed by the Tukey post hoc test were used to compare means between multiple groups in GraphPad Prism 6. (GraphPad Software, San Diego, CA, USA). A *p* value of 0.05 or lower was regarded as statistically significant.

## Figures and Tables

**Table 1:** The expression levels of differentially expressed proteins.

Gene Name	Up/Down-Regulated	Description	*p*-Value	FC
Ints4	Up	Integrator complex subunit 4	0.01973179	4.61333333
Rpl18	Up	60S ribosomal protein L18	0.03111818	2.18979266
Moxd1	Up	DBH-like monooxygenase protein 1	0.03943133	1.96772997
Rpl7	Up	60S ribosomal protein L7	0.0180495	1.9379361
Cadm2	Up	Cell adhesion molecule 2	0.04979333	1.76720858
Rps8	Up	40S ribosomal protein S8	0.0437712	1.69393939
Tubb5	Up	Tubulin beta-5 chain	0.04878117	1.65571049
Rpl34	Up	60S ribosomal protein L34	0.04677985	1.64879179
Bhmt	Up	Betaine--homocysteine S-methyltransferase 1	0.04297693	1.63417847
Cyfip1	Up	Cytoplasmic FMR1-interacting protein 1	0.04924497	1.60110533
Rpl7a	Up	60S ribosomal protein L7a	0.03507391	1.5979214
	Up	Uncharacterized protein C2orf72	0.0137668	1.45398773
Ctnna1	Up	Catenin alpha-1	0.0172758	1.41868198
Cdc42	Up	Cell division control protein 42	0.02439533	1.41036457
Adam9	Up	Disintegrin and metalloproteinase domain-containing protein 9	0.02993997	1.3840882
Atp2b1	Up	Plasma membrane calcium-transporting ATPase 1	0.03146976	1.37318303
Slc6a1	Up	Sodium- and chloride-dependent GABA transporter 1	0.00137502	1.34094235
Cnp	Up	2′,3′-cyclic-nucleotide 3′-phosphodiesterase	0.01201357	1.28534704
Nkain3	Up	Sodium/potassium-transporting ATPase subunit beta-1-interacting protein 3	0.03002822	1.2425007
Cask	Up	Peripheral plasma membrane protein CASK	0.02686701	1.23588597
Actr3	Down	Actin-related protein 3	0.03585085	0.82959744
Npc2	Down	NPC intracellular cholesterol transporter 2	0.03261727	0.80202703
Blmh	Down	Bleomycin hydrolase	0.04554183	0.79856115
Psph	Down	Phosphoserine phosphatase	0.01691792	0.79636282
Atp6v1a	Down	V-type proton ATPase catalytic subunit A	0.01888445	0.78930888
Mapre1	Down	Microtubule-associated protein RP/EB family member 1	0.04938624	0.77795066
Elavl1	Down	ELAV-like protein 1	0.0289941	0.7691287
Glo1	Down	Lactoylglutathione lyase	0.04542562	0.76422585
Atp6v1c1	Down	V-type proton ATPase subunit C 1	0.03806311	0.73310225
Plbd2	Down	Putative phospholipase B-like 2	0.00952826	0.73010381
Ddah1	Down	N(G),N(G)-dimethylarginine dimethylaminohydrolase 1	0.01486318	0.72043011
Hsp90aa1	Down	Heat shock protein HSP 90-alpha	0.0227131	0.70524297
Psmb2	Down	Proteasome subunit beta type-2	0.04881743	0.6939856
Ndufa4	Down	Cytochrome c oxidase subunit NDUFA4	0.03014898	0.65645061
Dctn1	Down	Dynactin subunit 1	0.02291716	0.65439504
Oat	Down	Ornithine aminotransferase	0.02557392	0.6400164
Rpn2	Down	Dolichyl-diphosphooligosaccharide--protein glycosyltransferase subunit 2	0.0427866	0.63445035
Ak3	Down	GTP:AMP phosphotransferase AK3	0.04882486	0.63211589
Psap	Down	Prosaposin	0.02450609	0.62423873
Hnrnpab	Down	Heterogeneous nuclear ribonucleoprotein A/B	0.03999623	0.61628612
Pdia3	Down	Protein disulfide-isomerase A3	0.0477473	0.57666535
Actg2	Down	Actin, gamma-enteric smooth muscle	0.01623375	0.50677966

## Data Availability

The proteomic data has been deposited to the ProteomeXchange Consortium (http://proteomecentral.proteomexchange.org) via the iProX partner repository with the dataset identifier PXD037966.

## Supplementary Materials

The following supporting information can be downloaded at: https://www.mdpi.com/article/10.3390/cimb45030127/s1, Figure S1: Negative control for neural stem cell identification; Table S1: Primer sequence.

## Abbreviations

CNS, central nervous system; EV, extracellular vesicle; NSC, neural stem cell; HP, hypoxic preconditioning; DEP, differentially expressed proteins.
