# Supplementary material for: Hypoxic Preconditioned Neural Stem Cell-Derived Extracellular Vesicles Contain Distinct Protein Cargo from Their Normal Counterparts
Source: Curr Issues Mol Biol. 2023 Mar 1;45(3):1982–97. doi: 10.3390/cimb45030127 (PMC10047917; doi:10.3390/cimb45030127)
Supplement: Supplementary file 1 [file cimb-45-00127-s001.zip › cimb-2209836-supplementary.pdf]

SUPPLEMENTARY MATERIALS FOR:

Hypoxic Preconditioned Neural Stem Cell Derived Extracellular Vesicles Contain Distinct Protein Cargo from Their Normal Counterparts

Tahereh Gharbi, Chang Liu, Haroon Khan, Zhijun Zhang, Guo-Yuan Yang * and Yaohui Tang *

Department of Biomedical Engineering, Shanghai Jiao Tong University, Shanghai 200025, China
Correspondence: gyyang@sjtu.edu.cn (G.-Y.Y.); yaohuitang@sjtu.edu.cn (Y.T.)

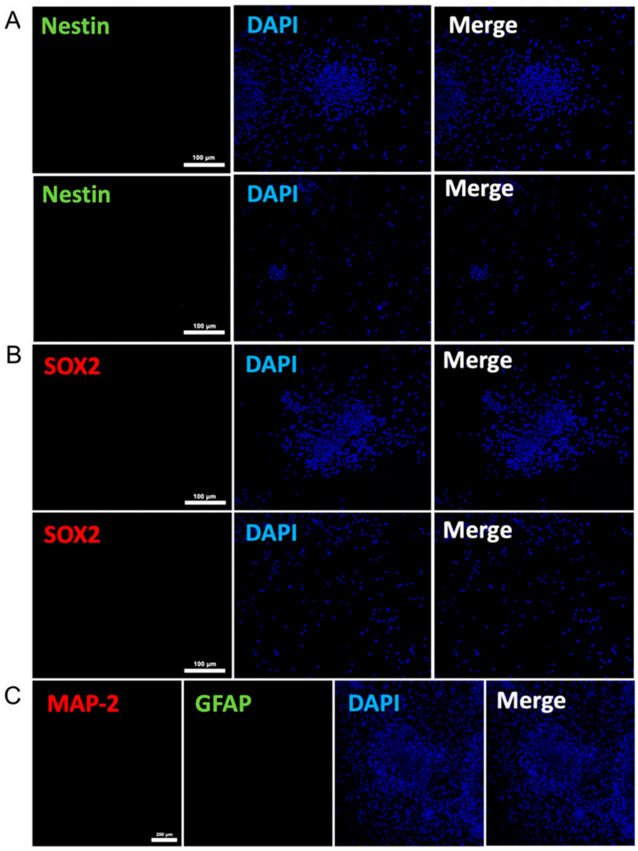

Figure S1. Negative control for neural stem cell identification: a-b) Negative control (no primary antibody) demonstrated no positive green and red staining, for both neurospheres (top row) and single cells (bottom row) for NSC markers of Nestin and SOX2. There is only DAPI positive staining (blue). **c)** Negative control (no primary antibody) showed no positive red staining for neural dendrites (MAP-2) and axons and no green staining for astrocytes (GFAP). There is only DAPI positive staining (blue).

Supplementary Table 1. Primer sequence

Primer	Sequence (5'-3')
Rpl34 mouse primer F	GCACCTAAATCTGCATGTGGCG
Rpl34 mouse primer R	TGTCACGGACACACTTGGCACA
Bhmt mouse primer F	GAGTTCCTCAGAGCTGGATCGA
Bhmt mouse primer R	TCATCAGCCACTTGCCGTGCAA
Cdc42 mouse primer F	GATTGGTGGAGAGCCATACACTC
Cdc42 mouse primer R	TGAGGATGGAGAGACCACTGAG
Atp2b1 mouse primer F	GCACAGTCTCAGAGCAACGACA
Atp2b1 mouse primer R	GCCACATCAGTTCCAGCAATGC
